# Enteroviral 2B Interacts with VDAC3 to Regulate Reactive Oxygen Species Generation That Is Essential to Viral Replication

**DOI:** 10.3390/v14081717

**Published:** 2022-08-04

**Authors:** Mei-Ling Cheng, Chien-Hsiang Wu, Kun-Yi Chien, Chien-Hsueh Lai, Guan-Jie Li, Yuan-Yu Liu, Gigin Lin, Hung-Yao Ho

**Affiliations:** 1 Department of Biomedical Sciences, College of Medicine, Chang Gung University, Taoyuan City 33302, Taiwan; 2 Healthy Aging Research Center, Chang Gung University, Taoyuan City 33302, Taiwan; 3 Metabolomics Core Laboratory, Healthy Aging Research Center, Chang Gung University, Taoyuan City 33302, Taiwan; 4 Clinical Metabolomics Core Laboratory, Chang Gung Memorial Hospital at Linkou, Taoyuan City 33302, Taiwan; 5 Graduate Institute of Biomedical Sciences, College of Medicine, Chang Gung University, Taoyuan City 33302, Taiwan; 6 Department of Medical Biotechnology and Laboratory Science, College of Medicine, Chang Gung University, Taoyuan City 33302, Taiwan; 7 Department of Biochemistry and Molecular Biology, College of Medicine, Chang Gung University, Taoyuan City 33302, Taiwan; 8 Department of Medical Imaging and Intervention, Chang Gung Memorial Hospital at Linkou, Taoyuan City 33302, Taiwan; 9 Imaging Core Laboratory, Institute for Radiological Research, Chang Gung University, Taoyuan City 33302, Taiwan; 10 Department of Medical Imaging and Radiological Sciences, Chang Gung University, Taoyuan City 33302, Taiwan; 11 Research Center for Emerging Viral Infections, Chang Gung University, Taoyuan City 33302, Taiwan

**Keywords:** EV71, VDAC3, reactive oxygen species

## Abstract

Enterovirus (EV) 71 caused episodes of outbreaks in China and Southeast Asia during the last few decades. We have previously reported that EV71 induces reactive oxygen species (ROS). However, the underlying mechanism remains elusive. Co-immunoprecipitation-proteomic analysis revealed that enteroviral 2B protein interacted with mitochondrial voltage-dependent anion channel 3 (VDAC3). Knockdown (KD) of *VDAC3* expression specifically inhibited enteroviral replication. Single-round viral replication was also inhibited in KD cells, suggesting that VDAC3 plays an essential role in replication. Consistent with this, *VDAC3* gene KD significantly reduced the EV71-induced mitochondrial ROS generation. Exogenous 2B expression could induce the mitochondrial ROS generation that was significantly reduced in *VDAC3*-KD cells or in the Mito-TEMPO-treated cells. Moreover, VDAC3 appears to be necessary for regulation of antioxidant metabolism. *VDAC3* gene KD led to the enhancement of such pathways as hypotaurine/taurine synthesis in the infected cells. Taken together, these findings suggest that 2B and VDAC3 interact to enhance mitochondrial ROS generation, which promotes viral replication.

## 1. Introduction

Enteroviruses caused a number of outbreaks in many parts of the world, leading to diseases such as poliomyelitis, Bornholm disease, myopericarditis, encephalitis, and many other clinical manifestations. Human enteroviruses fall in the genus *Enterovirus* of the family *Picornaviridae*, and are assigned to the species *Enterovirus A* to *D* (EV-A to D) and species *Rhinovirus A* to *C* (RV-A to C) [1]. Enterovirus has a ~7.5 kb-long single positive-strand RNA genome, whose recombination drives viral evolution. The genome encodes a polyprotein that is processed into structural proteins (VP1–VP4) and non-structural proteins (2A–2C and 3A–3D) [2,3]. EV71 and another strain, coxsackievirus A16, both of which are EV-A members, are pathogens for hand-foot-and-mouth disease (HFMD). The symptoms of HFMD include mild febrile illness and the presence of papulovesicular lesions on oral mucosa, extremities, and buttocks of the patients. Occasionally, infection with these viruses can cause encephalitis, aseptic meningitis, paralysis, and even fatality [4]. The largest outbreak of EV71 infection occurred in China in 2008 [5,6]. About 3 million cases with 1500 fatal cases were reported. 

Oxidative stress represents an imbalance between reactive oxygen species production and their removal by cellular antioxidant systems. Increase in oxidative stress is associated with changes in metabolism and physiology of host cells [7,8,9], and has a profound effect on viral infection. EV71 replication is enhanced in cells with increased oxidative stress [10]. In vivo infection with influenza virus depends on xanthine oxidase-mediated superoxide production [11]. A decrease in intracellular thiol content leads to activation of human immunodeficiency virus (HIV) [12]. Consistent with this, antioxidant treatment is inhibitory to viral replication. Injection of polymer-conjugated superoxide dismutase had a protective effect against influenza virus infection [13]. 

ROS generation increases significantly in response to infection with Sendai virus [14], human respiratory syncytial virus [15], rhinoviruses [16], influenza virus [17,18], and dengue virus [19]. We previously reported that EV71 infection induces ROS generation [10,20]. It has been recently reported that severe acute respiratory syndrome coronavirus 2 (SARS-CoV-2)-infected patients had increased levels of 2-thiobarbituric acid-reacting substances (TBARS) and F2-isoprostane, as well as glutathione (GSH) deficiency [21]. Mitochondria are an endogenous source of ROS. It has been found that hepatitis C virus and human immunodeficiency virus induce mitochondrial ROS generation via interaction between viral and host cell proteins [22,23]. We previously demonstrated that the EV71-induced ROS are of mitochondrial origin [20]. However, the mechanism underlying the EV71-induced mitochondrial ROS generation remains elusive. 

Voltage-dependent anion channels (VDACs) are pore-forming proteins localized to the outer membrane of mitochondria. In general, VDACs act as conduits for transport of metabolites and ions between cytoplasm and mitochondria. They are involved in such biochemical and physiological processes as regulation of energy metabolism, redox metabolism, and apoptosis [24]. Different isoforms have different mitochondrial localization and channel properties [25]. Sequence analysis of the cDNAs of three VDAC isoforms revealed 70% of identity [26]. Of three isoforms, VDAC3 is the least characterized one. Though the three-dimensional structure of VDAC3 has not been elucidated, its sequence similarity to VDAC1 and VDAC2 does contribute to prediction of its three-dimensional structure [25]. VDAC3 is thought to have a β-barrel, in which antiparallel β-strands are linked by short loops or turns. An N-terminal α-helix, composed of 25 amino acid residues, is postulated to reside in the lumen of the barrel and to constitute a gating mechanism [27]. Additionally, VDAC3 has six cysteine residues whose redox states may regulate its activity. Four of these cysteine residues face toward the mitochondrial intermembrane space [25]. 

EV71 2B protein is 99 amino acids long. It is a type II viroporin composed of two transmembrane helical regions, HR1 and HR2 [28]. The latter helices are connected by a stretch of polar amino acid residues. Both N- and C-termini protrude into the lumen of endoplasmic reticulum (ER) and Golgi body. Oligomerization of 2B gives rise to a proteinaceous channel with ion conductance [29,30,31]. For instance, expression of 2B was found to induce a transient rise in cytosolic calcium ion [32].

Viral infection is accompanied by the reprogramming of host cell metabolism [33]. Perturbation of redox metabolism occurs during viral metabolism. A significant increase in the oxidized glutathione (glutathione disulfide, GSSG) and a decrease in the reduced form, GSH, are accompanied by upregulation of glutathione biosynthesis in hepatitis C virus (HCV) [34]. We also found that the level of γ-glutamylcysteine, an intermediate in GSH synthesis, increases in EV71-infected cells [7]. 

In the present study, we demonstrate that the interaction between 2B and VDAC3 is essential to mitochondrial ROS generation and viral replication. It is also associated with reduction in production of taurine and hypotaurine. Silencing *VDAC3* gene that reduces the EV71-induced oxidative stress and enhances antioxidant metabolism is inhibitory to viral replication. These findings suggest that the 2B-VDAC3 interaction disrupts redox homeostasis in host cells to enhance viral replication. 

## 2. Materials and Methods

### 2.1. Cell Culture and Virus

Vero cells (ATCC CCL-81) and human rhabdomyosarcoma cells (RD; ATCC CCL-136) were cultured as described elsewhere [10,35]. The prototypic strain BrCr (ATCC VR-1775) was propagated as previously described [35]. 

### 2.2. Molecular Biology Techniques

The vector encoding the Myc-tagged GFP was previously described [7]. The vector expressing the Myc-tagged fusion of green fluorescent protein (GFP) and 2B (GFP-2B-Myc) was constructed by insertion of a 2B-coding fragment in between BspEI and HindIII of pcDNA3.1(-)/myc-His A.GFP. A loss-of-fluorescence mutation was created at C289A in the GFP coding sequence [36]. The resulting mutant protein was used in the experiments involving the use of fluorescent dyes. 

The vector encoding N- or C-terminally FLAG-tagged VDAC3 was constructed as follows. RNA was extracted from RD cells using TRIzol (Thermo Fisher Scientific, Waltham, MA, USA), and reverse-transcribed to cDNA with RevertAid RT Reverse Transcription Kit (Thermo Fisher Scientific). The *VDAC3* cDNA was amplified with forward primer (5′-TGTAACACACCAACGTACTGTGACCTAGGAAAGGC-3′) and reverse primer (5′-CAATTGTTAAGCTTCCAGTTCAAATCCCAAGCCAACCTTG-3′), and was cloned in PsiI and MfeI sites of the vector pQCXIP-3×Flag to generate pQC-XIP.n3×Flag.VDAC3. The DNA fragment encoding N-terminally FLAG-tagged VDAC3 protein was cloned in between XhoI and BamHI sites of pcDNA3.1(-)/myc-His A vector. For construction of the vector encoding C-terminally FLAG-tagged VDAC3, the *VDAC3* cDNA was amplified with forward primer (5′-AACCTCGAGGCCACCATGTGTAACACACCAACGTACTGTGACCTAGGAAAGG-3′) and reverse primer (5′-GGCAGCTTCCAGTTCAAATCCCAAGCCAACCTTGTGACCT-3′). It was cloned between XhoI and NaeI sites of the vector pQCXIP-3×Flag to create pQC-XIP.c3×Flag.VDAC3, and subsequently sub-cloned in pcDNA3.1(-)/myc-His A vector. Expression of both N- and C-terminally FLAG-tagged VDAC3 was tested with the cell lines used. Only N-terminally FLAG-tagged VDAC3 was efficiently expressed. The expression vector encoding the N-tagged protein was used in subsequent experiments. A control vector pC34V expresses a fusion protein of Cerulean protein and Venus protein C34V, which are connected by a linker with an NIa protease recognition sequence and a FLAG epitope [37].

JetPRIME (Polyplus; Illkirch, France) was used for transfection according to manufacturer’s instruction. 

The lentiviral vectors encoding the shRNAs against *VDAC3* gene (TRCN0000139639) and that encoding non-target control (NTC) shRNAs were acquired from RNA Technology Platform and Gene Manipulation Core Laboratory (Academia Sinica, Taipei, Taiwan). One μg of each vector was co-transfected with 2.25 μg of pCMV-ΔR8.91 and 0.25 μg of pMD.G into 293-FT cells according to core laboratory protocols. The supernatant containing lentiviral particles was harvested at 24 and 48 h after transfection, filtered through 0.45 μm syringe filter, and stored at −80 °C. For transduction, about 10^5^ Vero or RD cells were seeded in 60 mm plate. The virus-containing supernatant was mixed with 8 μg/mL polybrene, and added to Vero or RD cells. Forty-eight hours later, the transduced cells were selected in the presence of 2 μg/mL puromycin. 

The reverse-transcription quantitative polymerase chain reaction (RT-qPCR) was performed as previously described [38]. The forward primer for quantification of *VDAC3* expression was 5′-TGCGGACTTCCAGCTGCACACA-3′; the reverse primer used was 5′-CCCAGTCCAATCAGGCTGGCATT-3′. 

### 2.3. Virological Techniques

Plaque formation and EV71 genomic RNA quantification were performed as described elsewhere [38,39]. In general, infection of Vero, RD, or their derived cells with EV71 at an indicated multiplicity of infection (MOI) was allowed to take place for 1 h at 37 °C. 

### 2.4. Immunoprecipitation and Proteomic Analysis

About 5 × 10^5^ Vero cells were transfected with 1 μg of expression vector encoding GFP-2B-Myc (pcDNA3.1(-)/myc-His A.GFP.2B) or 0.2 μg of expression vector encoding GFP-Myc (pcDNA3.1(-)/myc-His A.GFP) (plus 0.8 μg of pcDNA3.1(-)/myc-His A) using JetPRIME. Cells were harvested for immunoprecipitation with Myc monoclonal antibody (9E10) (Thermo Fisher Scientific) bound to Dynabead Protein G magnetic beads (Thermo Fisher Scientific) as described previously [7]. Protein was eluted from beads with 0.1% trifluoroacetic acid/50% acetonitrile. The eluate was lyophilized and processed for proteomic analysis as described [7]. The mass spectrometer was run in positive ion mode, and the full scan spectra (with *m/z* ranging from 400 to 2000 and a resolution of 60,000) were acquired by orbitrap analyzer. The tandem mass spectrometric (MS/MS) analysis was performed by the ion trap. For each spectrum, top 15 precursor ions whose ion intensities were greater than 5000 were processed for MS/MS analysis. The MS data were analyzed with Proteome Discoverer software (version 1.3.0.339). The MS/MS spectra were searched against the Swiss-Prot human database (version 2010_6) with the help of Mascot search engine (version 2.2; Matrix Science Inc., Boston, MA, USA). Proteins that were identified with at least two unique peptides were confident identifications, and quantified by determining the median of ratios of the corresponding unique peptides. Any proteins that specifically co-immunoprecipitated with GFP-2B-Myc versus with GFP-Myc represent those host cell proteins interacting with 2B. Ingenuity pathway analysis (IPA) was used to analyze the proteins differentially co-immunoprecipitated with GFP-2B-Myc versus GFP-Myc. 

### 2.5. Immunological Techniques

To study the interaction between 2B and VDAC3, we transfected Vero cells with vectors encoding GFP-2B-Myc and N-FLAG-VDAC3. Immunoprecipitation was carried out with Dynabead Protein G magnetic beads that had been pre-coated with Myc monoclonal antibody (9E10) or FLAG M2 monoclonal antibody (Sigma-Aldrich, St. Louis, MI, USA) as described elsewhere [7]. 

For analysis of the immunoprecipitated proteins, the protein-bound beads were mixed with Laemmli sample buffer and heated at 95 °C for 5 min. The sample was analyzed by Western blotting as previously described [38]. The antibody to actin (Clone AC-40) was purchased from Sigma-Aldrich. A horseradish peroxidase (HRP)-conjugated secondary antibody to mouse IgG (SC-2005) was available from Santa Cruz Biotechnology Inc. (Dallas, TX, USA). The HRP-conjugated anti-Myc antibody (R951-25) and anti-FLAG antibody (MA1-91878) were purchased from Thermo Fisher Scientific. 

### 2.6. Confocal Microscopy

Cells were cultured in glass-bottomed culture dish (Nunc, Thermo Fisher Scientific), and transfected with 0.5 μg pcDNA3.1(-)/myc-His A.GFP.2B plus 1 μg pcDNA3.1(-)/myc-His A; 1 μg pcDNA3.1(-)/myc-His A.n3×Flag.VDAC3 plus 0.5 μg pcDNA3.1(-)/myc-His A; or 0.5 μg pcDNA3.1(-)/myc-His A.GFP.2B plus 1 μg pcDNA3.1(-)/myc-His A.n3×Flag.VDAC3. Twenty-four hours later, the transfected cells expressing GFP-2B-Myc were stained with 100 nM MitoTracker Red for 20 min at 37 °C, and counterstained with 5 μg/mL of Hoechst 33342. This was followed by confocal microscopic examination. The transfected cells expressing N-FLAG-VDAC3 were stained with MitoTracker Red and Hoechst 33342 as described above. The cells were subsequently fixed in 2% paraformaldehyde for 15 min, and permeabilized with phosphate buffered saline (PBS) containing 0.1% Triton X-100 (PBST). The specimens were then blocked with PBST/2% bovine serum albumin (BSA), and incubated with fluorescein isothiocyanate (FITC)-conjugated anti-FLAG antibody (F4049; Sigma-Aldrich) at a dilution of 1:1000. After several rinses with PBST, the specimens were subjected to confocal microscopic examination. Likewise, the transfected cells expressing N-FLAG-VDAC3 and GFP-2B-Myc were fixed and permeabilized as described above. The specimens were incubated with Cy3-anti-FLAG antibody (A9594; Sigma-Aldrich) at a dilution of 1:1000. After washes, they were examined using confocal microscope.

For confocal microscopic examination, Zeiss LSM 510 Meta system (Carl Zeiss Microscopy, Oberkochen, Germany) was used. Fluorescence images of the stained cells were acquired with a Plan-Apochromat 100 × 1.40 NA oil immersion objective. For scanning of the GFP or FITC fluorescence signal, an argon laser (488 nm excitation line), a beam splitter (HFT 405/488/561/633/KP720), and an emission window (505–550 nm) were used. For scanning of MitoTracker Red and Cy3 fluorescence signal, an excitation line of DPSS (561 nm) and an emission window (575–615 nm) were employed. For scanning of the Hoechst dye fluorescence signal, an excitation line (405 nm) of diode laser and an emission window (420–480 nm) were used. All images were then analyzed using Zeiss Zen software. 

### 2.7. Cytometric Analysis

For ROS quantification, cells were mock infected or infected with EV71 for 24 h, and subsequently stained with 2.5 μM CellROX Deep Red at 37 °C for 30 min. The cell monolayer was dissociated into single-cell suspension, and after washing, resuspended in PBS. The fluorescence in FL4 channel was measured by FACScalibur flow cytometer (Beckton Dickenson Biosciences, Franklin Lakes, NJ, USA). For quantification of mitochondrial ROS generation, cells were similarly infected and loaded with 2.5 μM MitoSOX Red. The fluorescence of the labeled cells was measured in FL2 channel by the flow cytometer. 

### 2.8. Metabolite Analysis

About 10^6^ cells were cultured in 100 mm culture dish, and infected with EV71 at an indicated MOI for 1 h. An equal volume of MEM/2% FCS was added. Sixteen hours later, the cells were harvested in 80% methanol/0.1% formic acid solution as described previously [7]. The extract was dried under nitrogen gas and dissolved in 0.1% formic acid. The sample was analyzed using liquid chromatography time-of-flight mass spectrometry (LC-TOF-MS) as described elsewhere [7]. Spectral data were analyzed with Progenesis QI software (Waters Corp., Milford, MA, USA) for peak peaking, alignment, and normalization. The features were identified through Metabolite Link (METLIN) and Human Metabolome (HMDB) database search, and/or through comparison to the chromatographic and spectral data of standard compounds. 

### 2.9. Statistical Analysis

All statistical analyses were performed using GraphPad Prism 7 software (GraphPad Software Inc., San Diego, CA, USA). Normal distribution of the data was evaluated by Shapiro–Wilk test. Two-tailed unpaired Student’s *t* test, two-way analysis of variance (ANOVA) with Sidak’s multiple-comparison test, Kruskal–Wallis test with Dunn’s multiple comparison test, and Mann–Whitney test were applied where appropriate. 

## 3. Results

### 3.1. Reduction in EV71-Induced ROS Generation Is Inhibitory to Viral Replication

**Figure 1:**
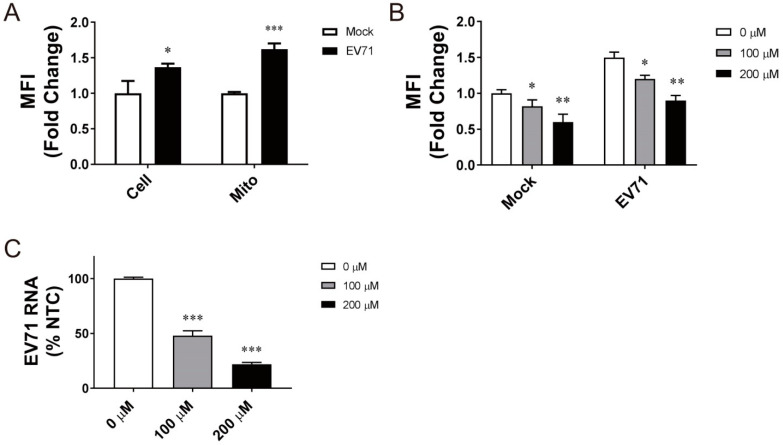
Mitochondrial ROS generation essential to EV71 replication. (**A**) Vero cells were mock infected (*Mock*) or infected (*EV71*) with BrCr virus at an MOI of 1.25, and stained with CellROX Deep Red for quantification of total cellular ROS (*Total*) or with MitoSOX Red for quantification of mitochondrial ROS (*Mito*) at 24 h post-infection. The MFI is expressed as fold change relative to that of mock-infected cells. The results are mean ± SD, *n* = 6. * *p* < 0.05, *** *p* < 0.005 vs. mock-infected cells. (**B**) Vero cells were mock infected (*Mock*) or infected (*EV71*) with BrCr virus at an MOI of 1.25; treated without or with 100 μM and 200 μM Mito-TEMPO; and stained with MitoSOX Red at 24 h post-infection. The MFI is expressed as fold change relative to that of untreated mock-infected cells. The results are mean ± SD, *n* = 6. * *p* < 0.05, ** *p* < 0.01 vs. mock-infected cells. (**C**) Cells were mock infected (*Mock*) or infected (*EV71*) with BrCr virus at an MOI of 1.25; treated without or with 100 μM and 200 μM Mito-TEMPO; and extracted for determination of EV71 genomic RNA. The RNA level is expressed as the percentage of that of untreated infected cells. The results are mean ± SD, *n* = 6. *** *p* < 0.005 vs. infected cells.

EV71 replication is accompanied by ROS generation. Vero cells were infected with EV71 and stained with CellROX Deep Red or MitoSOX Red. As shown in Figure 1A, the mean fluorescence intensity (MFI) of the CellROX Deep Red- or MitoSOX Red-stained cells increased significantly, suggesting that the mitochondrial ROS generation is induced by EV71. To study if the mitochondrial ROS production is essential to viral replication, we treated the EV71-infected cells with mitochondrion-specific antioxidant Mito-TEMPO, and studied its effect on viral replication. Mito-TEMPO treatment suppressed mitochondrial ROS production in mock- and EV71-infected cells in a concentration-dependent manner (Figure 1B). Treatment with 100 μM and 200 μM Mito-TEMPO significantly reduced the level of EV71 RNA by 53% and 78%, respectively (Figure 1C). Mito-TEMPO was not cytotoxic at the concentrations used (Figure S1A). These findings suggest that the mitochondrial ROS formation is essential to viral replication. 

### 3.2. Identification of Protein-Protein Interaction That Is Important in Mitochondrial ROS Generation 

**Figure 2:**
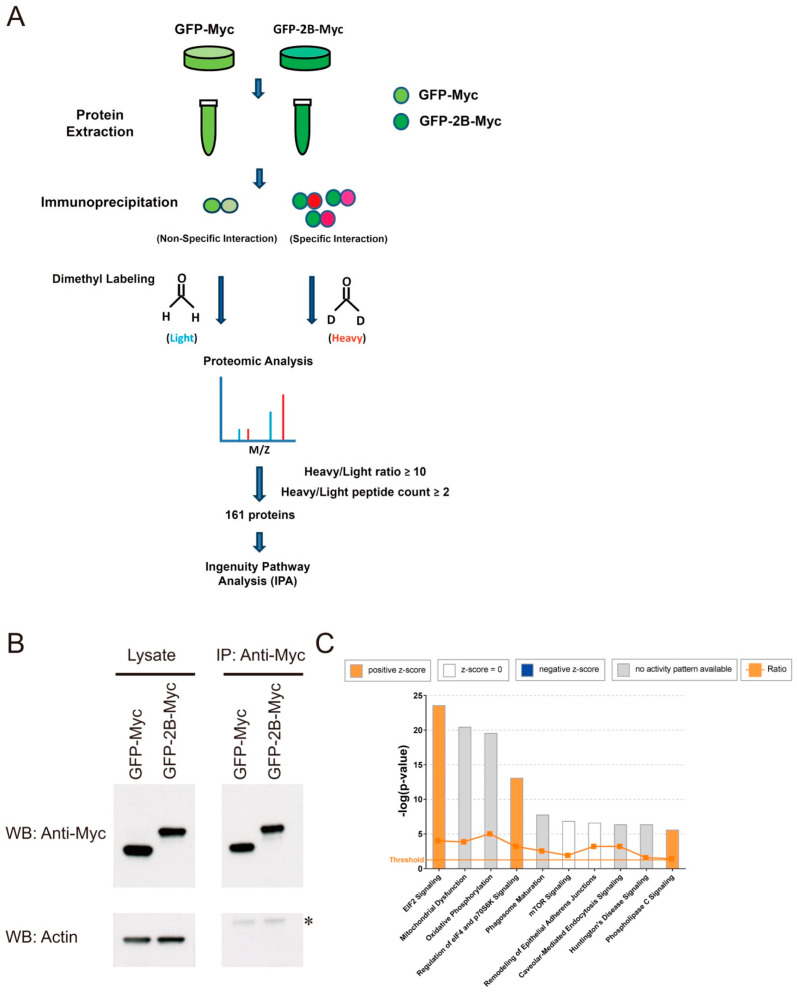
Identification of the 2B-interacting proteins through immunoprecipitation-mass spectrometry. (**A**) Vero cells were transfected with vectors encoding GFP-Myc (control) or GFP-2B-Myc proteins, and lysates were prepared. Immunoprecipitation with the anti-Myc antibody was followed by tryptic digestion and labeling with light (hydrogen) and heavy (deuterium) stable-isotope dimethyl formaldehyde. Peptides were analyzed by the strong cation exchange (SCX)/reverse-phase nanoscale liquid chromatography (LC) coupled with Orbitrap Elite hybrid mass spectrometer. The proteins with heavy/light ratio ≥ 10 and heavy/light count ≥ 2 were identified, and analyzed using IPA. (**B**) Cell lysate (*Lysate*) and the anti-Myc (*Anti-Myc*) immunoprecipitate (*IP*) were analyzed by Western blotting (*WB*) for the presence of GFP-Myc, GFP-2B-Myc and actin. The asterisk (*) indicates a band cross-reactive with the particular batch of anti-actin antibody. A representative result of three experiments is shown here. (**C**) The proteome dataset was analyzed using IPA, and the top canonical pathways are shown. The horizontal line shows the threshold for *p*-value. The bars are color coded according to their *z*-score or the absence of activity pattern.

We adopted a proteomic approach to identify any viral proteins that interact with host cell proteins and regulate redox metabolism (Figure 2A). A number of vectors coding for the c-Myc-tagged fusion proteins between GFP and EV71 proteins were constructed. Vero cells were transfected with vectors encoding these fusion proteins and c-Myc-tagged GFP (control). Immunoprecipitation was carried out with anti-Myc antibody, and the co-immunoprecipitated proteins were identified. As will be mentioned later, expression of 2B is associated with an increased mitochondrial ROS generation. Expression of GFP-2B-Myc and GFP-Myc for the immunoprecipitation–mass spectrometry study is shown (Figure 2B). The proteins differentially co-immunoprecipitated with GFP-2B-Myc were analyzed using IPA. For this dataset, top 10 canonical pathways that have –log(*p*-value) above threshold are shown (Figure 2C). These pathways included EIF2 signaling; mitochondrial dysfunction; oxidative phosphorylation; regulation of eIF4 and p70S6K signaling; phagosome maturation; mTOR signaling; remodeling of epithelial adherens junctions; caveolar-mediated endocytosis signaling; Huntington’s disease signaling; and phospholipase C signaling. Of these pathways, the mitochondrial dysfunction pathway is particularly associated with the EV71-induced ROS generation [9]. A number of mitochondrial proteins were found in this category, and the top 10 differentially abundant co-immunoprecipitated proteins are shown (Table S1). One of these proteins is VDAC3. 

### 3.3. Interaction between 2B and VDAC3

**Figure 3:**
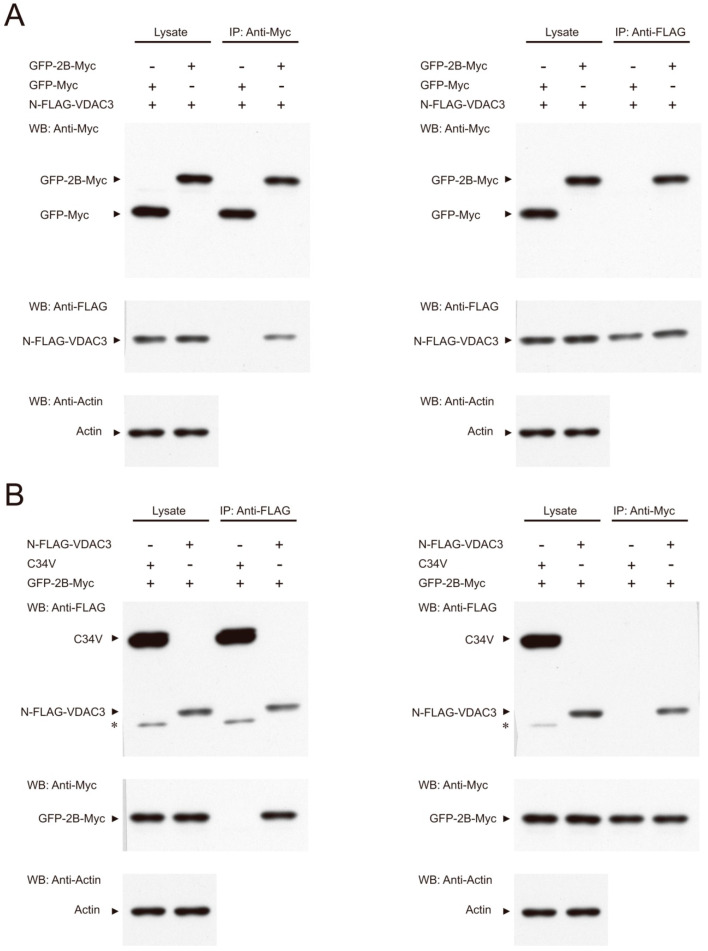
Interaction between 2B and VDAC3. (**A**) Vero cells were transfected with GFP-2B-Myc expression vector (or the one expressing the control protein GFP-Myc) and N-FLAG-VDAC3 expression vector. Cell lysate was prepared for immunoprecipitation (*IP*) with anti-Myc (left panel) or anti-FLAG (right panel) antibody. The cell lysate and immunoprecipitate were analyzed by Western blotting with antibody to anti-Myc epitope tag, anti-FLAG epitope tag, or anti-actin (for total cell lysate). (**B**) Vero cells were transfected with N-FLAG-VDAC3 expression vector (or the expression vector encoding the FLAG-tagged C34V control protein) in addition to GFP-2B-Myc expression vector. Cells were lysed for immunoprecipitation (*IP*) with anti-FLAG (left panel) or anti-Myc (right panel) antibody. The cell lysate and immunoprecipitate were analyzed by Western blotting with antibody to anti-Myc epitope tag, anti-FLAG epitope tag, or anti-actin (for total cell lysate). The asterisk (*) indicates a degradation product of C34V. A representative result of three experiments is shown here.

The interaction between 2B and VDAC3 was validated by immunoprecipitation. Vero cells were transfected with the GFP-2B-Myc expression vector (or the GFP-Myc expression vector as control) in addition to the N-FLAG-VDAC3 expression vector. Immunoprecipitation with the anti-Myc antibody demonstrated that GFP-2B-Myc, but not GFP-Myc, co-immunoprecipitated N-FLAG-VDAC3 (Figure 3A, left panel). In a reciprocal manner, immunoprecipitation with the anti-FLAG antibody showed that N-FLAG-VDAC3 specifically pulled down GFP-2B-Myc (Figure 3A, right panel). Moreover, transfection of Vero cells with the GFP-2B-Myc expression vector and the N-FLAG-VDAC3 expression vector (or the C34V expression vector) was performed. Immunoprecipitation with the anti-FLAG antibody showed that N-FLAG-VDAC3, but not C34V protein, co-immunoprecipitated GFP-2B-Myc. Reciprocal immunoprecipitation with the anti-Myc antibody revealed that GFP-2B-Myc specifically bound N-FLAG-VDAC3 (Figure 3B). These findings suggest that VDAC3 interacts with 2B in a specific manner. 

**Figure 4:**
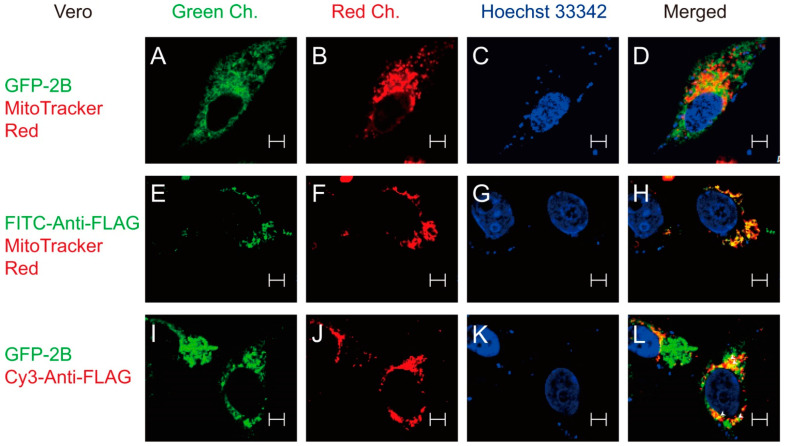
Localization of 2B and VDAC3 to the periphery of mitochondria. (**A**–**D**) Vero cells were transfected with the GFP-2B expression vector, and stained with MitoTracker Red and Hoechst dye 33342 at 24 h post-transfection. They were counterstained with Hoechst 33342. The samples were processed for confocal microscopic examination of 2B (*Green Channel*), mitochondria (*Red Channel*), and nuclei (*Hoechst 33342*). The images are overlaid (*Merged*). (**E**–**H**) Vero cells were transfected with N-FLAG-VDAC3 expression vector, and stained with FITC-conjugated anti-FLAG antibody and MitoTracker Red at 24 h post-transfection. They were counterstained with Hoechst 33342. The samples were processed for confocal microscopic examination of FITC-conjugated anti-FLAG antibody-bound VDAC3 (*Green Channel*), mitochondria (*Red Channel*), and nuclei (*Hoechst 33342*). The images are overlaid (*Merged*). (**I**–**L**) Vero cells were transfected with GFP-2B and N-FLAG-VDAC3 expression vectors, and stained with Cy3-conjugated anti-FLAG antibody at 24 h post-transfection. They were counterstained with Hoechst 33342. The samples were processed for confocal microscopic examination of 2B (*Green Channel*), VDAC3 (*Red Channel*), and nuclei (*Hoechst 33342*). The white arrowheads indicate the specific locations at which 2B and VDAC3 bind. The images are overlaid (*Merged*). A representative result of three experiments is shown here. Scale bar = 5 μm.

To further study the interaction between 2B and VDAC3, we studied the subcellular localization of GFP-2B and/or N-FLAG-VDAC3. Staining of the GFP-2B-expressing Vero cells with MitoTracker Red showed that the GFP-2B fluorescence signal was found to overlap with the MitoTracker Red fluorescence signal at the periphery of mitochondria in a speckled manner (Figure 4A–D). Expression of N-FLAG-VDAC3, followed by immunofluorescence staining and MitoTracker Red staining, revealed a complete overlap in the fluorescence signals of VDAC3 and mitochondria (Figure 4E–H). This is consistent with the subcellular localization of VDAC3 [40]. Expression of GFP-2B and N-FLAG-VDAC, which was followed by immunofluorescence staining, indicated that the GFP-2B fluorescence signal coincided with the Cy3 fluorescence signal of antibody-stained N-FLAG-VDAC3 at discrete locations (Figure 4I–L). These findings suggest that 2B interacts with VDAC3 at specific positions on the periphery of mitochondria. 

### 3.4. Specific Role of VDAC3 in EV71 Replication

**Figure 5:**
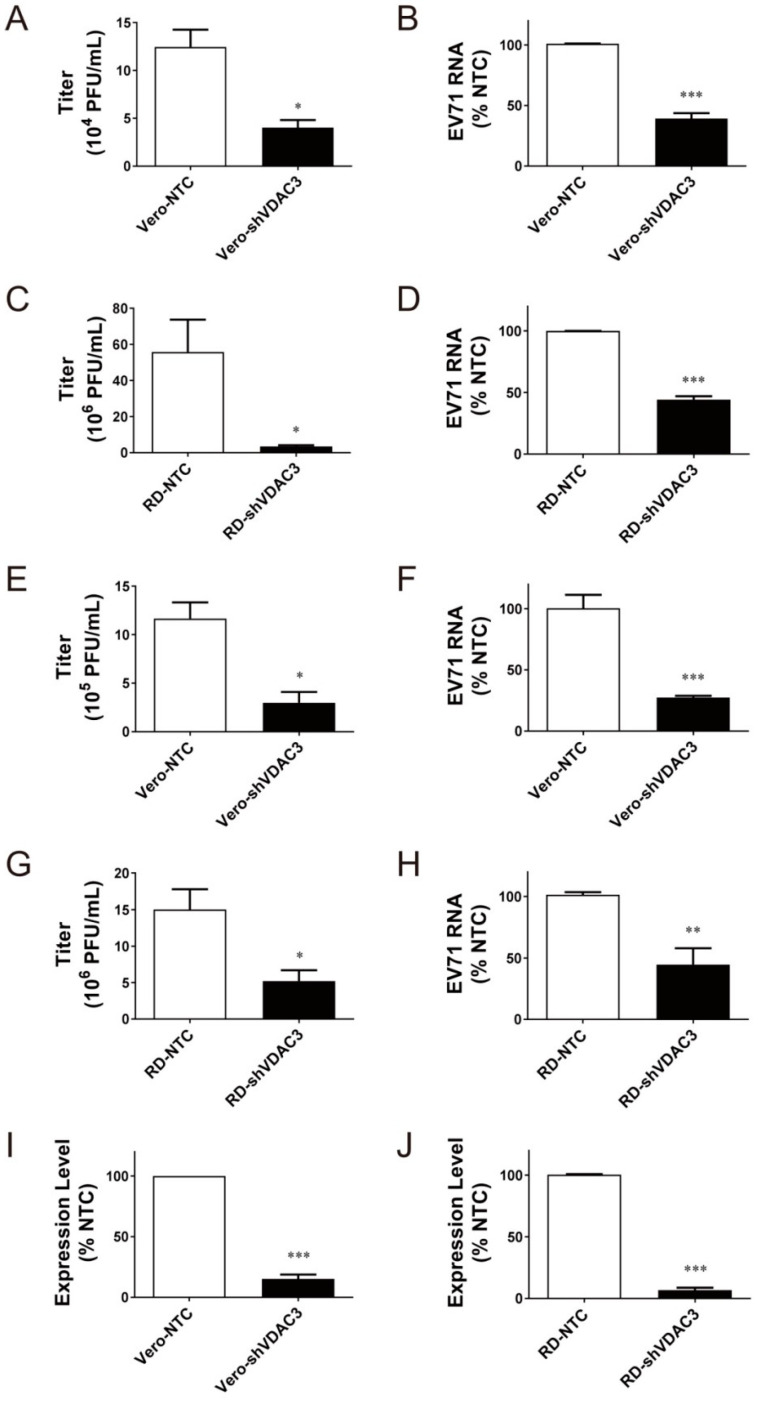
VDAC3 plays an essential role in viral replication. Vero and RD cells were transduced with the lentiviral vectors encoding control shRNA (*NTC*), or the shRNA against VDAC3 (*shVDAC3*), to generate Vero-NTC, Vero-shVDAC3, RD-NTC, and RD-shVDAC3 cells, respectively. (**A**–**D**) Vero-shVDAC3 and Vero-NTC cells were infected with EV71 at an MOI of 1.25 for 1 h (**A**,**B**), while RD-shVDAC3 and RD-NTC cells were infected at MOI of 0.01 (**C**,**D**). Twenty-four hours later, the titer (**A**,**C**) and genomic RNA level (**B**,**D**) of the progeny virus were determined. (**E**–**H**) Vero-shVDAC3 and Vero-NTC cells were infected with EV71 at an MOI of 10 for 1 h (**E**,**F**). RD-shVDAC3 and RD-NTC cells were similarly infected (**G**,**H**). Six hours later, the titer (**E**,**G**) and genomic RNA level (**F**,**H**) of the progeny virus were determined. (**I**,**J**) The levels of *VDAC3* transcript in Vero-shVDAC3 and Vero-NTC cells (**I**), and RD-shVDAC3 and RD-NTC cells (**J**) were quantified by RT-qPCR. The level of *VDAC3* transcript is expressed as the percentage of that of respective NTC cells. The results are mean ± SD, *n* = 6. * *p* < 0.05, ** *p* < 0.01, *** *p* < 0.005 vs. respective NTC cells.

To study if VDAC3 is involved in EV71 replication, we derived Vero and RD cells expressing VDAC3-specific shRNA. The derived cells were named Vero-shVDAC3 and RD-shVDAC3, respectively. As control, Vero and RD cells expressing NTC shRNA were also derived, and named Vero-NTC and RD-NTC, respectively. Vero-shVDAC3 and Vero-NTC cells were infected with EV71 at MOI of 1.25 for 1 h, and the extent of viral replication was determined at 24 h post-infection. Knockdown of *VDAC3* expression reduced both titer (Figure 5A) and genomic RNA (Figure 5B) of the progeny virus. Similar results were obtained for RD-shVDAC3 cells (and RD-NTC cells) that were infected at an MOI of 0.01 and incubated for 24 h (Figure 5C,D). Moreover, we studied the effect of shVDAC3 on single-round viral replication. Vero-shVDAC3 and Vero-NTC cells were infected with EV71 at an MOI of 10 for 1 h, and the extent of viral replication was determined at 6 h after infection. Both viral titer and genomic RNA level decreased substantially in Vero-shVDAC3 cells, as compared with Vero-NTC cells (Figure 5E,F). Similar results were observed for RD-shVDAC3 cells (and RD-NTC cells) (Figure 5G,H). The specificity of involvement of VDAC3 in replication was shown by failure of the shRNAs against VDAC1 (shVDAC1) and TOMM70A (shTOMM70A) to inhibit viral replication in Vero cells (Figure S2). Interestingly, expression of shTOMM70A did not inhibit but instead increased the viral replication (Figure S2B). All these findings suggest that VDAC3 plays an essential role in viral replication.

### 3.5. Interaction between 2B and VDAC3 in EV71-Induced Mitochondrial ROS Generation

**Figure 6:**
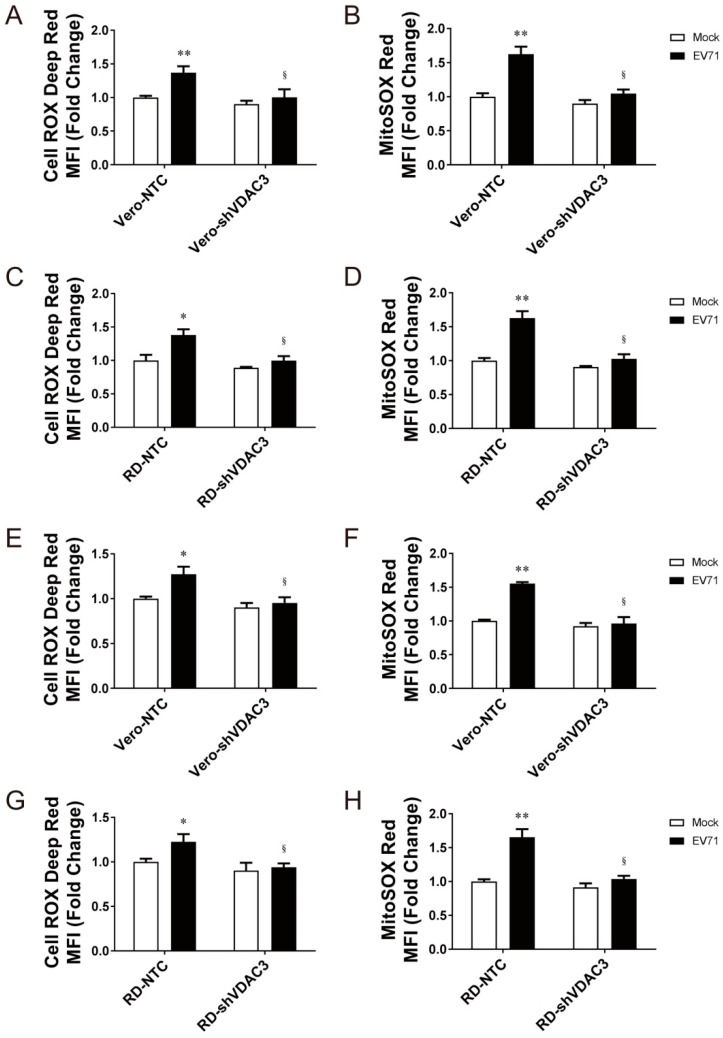
VDAC3 is essential to mitochondrial ROS generation. (**A**–**D**) Vero-shVDAC3 and Vero-NTC cells (**A**,**B**) were mock infected or infected with EV71 virus at an MOI of 1.25. RD-shVDAC3, and RD-NTC cells (**C**,**D**) were mock infected or infected with EV71 virus at an MOI of 0.01. Twenty-four hours later, the cells were stained with CellROX Deep Red (**A**,**C**) for quantification of total cellular ROS, or with MitoSOX Red (**B**,**D**) for quantification of mitochondrial ROS. (**E**–**H**) Vero-shVDAC3 and Vero-NTC cells (**E**,**F**), or RD-shVDAC3 and RD-NTC cells (**G**,**H**) were mock infected or infected with EV71 virus at an MOI of 10. Six hours later, the cells were stained with CellROX Deep Red (**E**,**G**) for quantification of total cellular ROS or with MitoSOX Red (**F**,**H**) for quantification of mitochondrial ROS. The MFI is expressed as fold change relative to that of mock-infected NTC cells. The results are mean ± SD, *n* = 6. * *p* < 0.05, ** *p* < 0.01 vs. mock-infected NTC cells; ^§^ *p* < 0.05 vs. infected NTC cells.

To study the role of VDAC3 in virus-induced mitochondrial ROS generation, we mock infected or infected Vero-shVDAC3 cells, RD-shVDAC3 cells, and their NTC-expressing counterparts with EV71, and determined total cellular and mitochondrial ROS formation at 24 h post-infection (Figure 6A–D). Silencing of *VDAC3* expression significantly reduced the virus-induced increase in the CellROX Deep Red and MitoSOX Red fluorescence of the stained cells. Similarly, the fluorescence of stained Vero-shVDAC3 and RD-shVDAC3 cells decreased within one round of viral replication, as compared with that of their NTC counterparts (Figure 6E–H). These findings suggest that VDAC3 is essential to induction of mitochondrial ROS formation in the infected cells.

**Figure 7:**
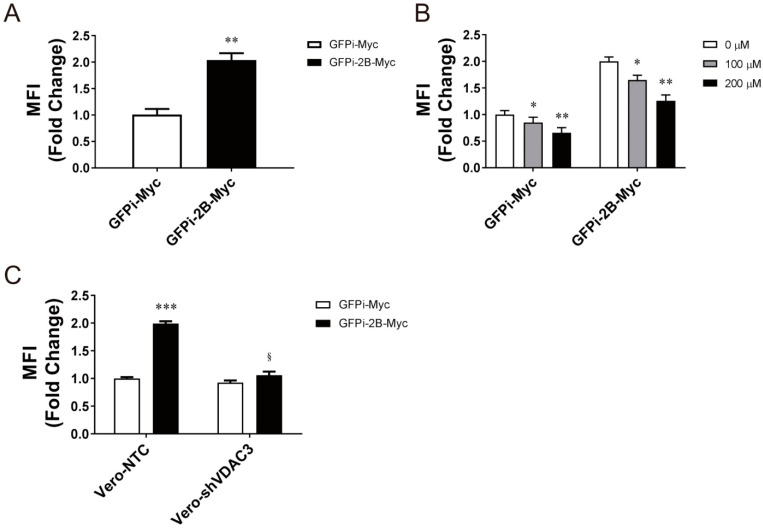
2B interacts with VDAC3 to induce mitochondrial ROS generation. (**A**) Vero cells were transfected with the GFPi-2B-Myc or GFPi-Myc expression vector. Forty-eight hours later, the cells were stained with MitoSOX Red for quantification of mitochondrial ROS. The results are mean ± SD, *n* = 6. ** *p* < 0.01 vs. the GFPi-Myc-expressing cells. (**B**) Vero cells were transfected as described above, and treated without or with 100 μM and 200 μM Mito-TEMPO at 24 h after transfection. Twenty-four hours later, the cells were stained with MitoSOX Red for quantification of mitochondrial ROS. The results are mean ± SD, *n* = 6. * *p* < 0.05, ** *p* < 0.01 vs. corresponding untreated cells. (**C**) Vero-shVDAC3 and Vero-NTC cells were transfected with the GFPi-2B-Myc or GFPi-Myc expression vector. Forty-eight hours later, the cells were stained with MitoSOX Red for quantification of mitochondrial ROS. The results are mean ± SD, *n* = 6. *** *p* < 0.01 vs. Vero-NTC cells; ^§^ *p* < 0.05 vs. the GFPi-2B-Myc-expressing Vero-NTC cells.

To examine if interaction between 2B and VDAC3 enhances mitochondrial ROS production, we transfected Vero cells with the expression vector encoding the Myc-tagged fusion protein between non-fluorescent GPF (GFPi) and 2B (GFPi-2B-Myc) or the control protein GFPi-Myc, and determined the level of mitochondrial ROS in the transfected cells. As shown in Figure 7A, expression of 2B increased the MitoSOX Red fluorescence increased by nearly twofold. However, the fluorescence intensity diminished upon treatment with Mito-TEMPO (Figure 7B). We further studied if VDAC3 is essential to mitochondrial ROS generation. We transfected Vero-shVDAC3 and Vero-NTC cells with the expression vector encoding GFPi-2B-Myc or GFPi-Myc, and quantified mitochondrial ROS. Expression of 2B significantly increased MitoSOX Red fluorescence in Vero-NTC cells but not in Vero-shVDAC3 cells (Figure 7C), suggestive of an essential role of VDAC3 in 2B-induced mitochondrial ROS formation. These findings suggest that 2B interacts with VDAC3 to regulate mitochondrial ROS generation.

### 3.6. EV71 Infection Is Associated with Changes in Antioxidant Metabolism

**Figure 8:**
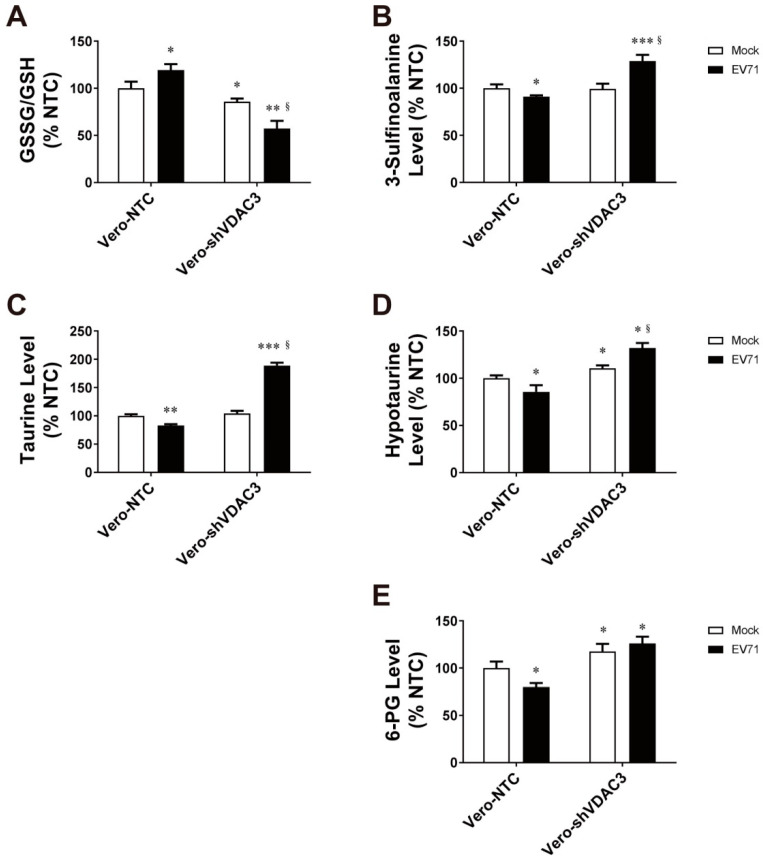
Alterations in antioxidant metabolism in EV71-infected cells. Vero-NTC and Vero-shVDAC3 cells were mock infected (*Mock*) or infected (*EV71*) at an MOI of 1.25, and extracted at 16 h post-infection for metabolomic analysis with UPLC-Q-TOF-MS (*n* = 18). Their GSSG/GSH ratio (**A**) and intracellular levels of 3-sulfinoalanine (**B**), taurine (**C**), hypotaurine (**D**) and 6-PG (**E**) are shown. The GSSG/GSH ratio and the levels of metabolites are expressed as a percentage of those of the mock-infected Vero-NTC cells. Data are mean ± SD (*n* = 18). * *p* < 0.05, ** *p* < 0.01, *** *p* < 0.005, vs. mock-infected Vero-NTC cells; ^§^ *p* < 0.05 vs. infected Vero-NTC cells.

We have previously demonstrated that EV71 induces metabolic reprogramming in host cells. As virus-induced oxidative stress diminishes in the *VDAC3*-KD cells, we proceeded to studying the antioxidants in the infected Vero-NTC and Vero-shVDAC3 cells. These cells were infected with EV71 at an MOI of 1.25, and extracted for metabolite analysis at 16 h post-infection. EV71 infection resulted in an increase in the ratio of glutathione disulfide (GSSG) to glutathione (GSH) in Vero-NTC cells, which was indicative of an increased oxidative stress. Intriguingly, the GSSG/GSH ratio was lowered in Vero-shVDAC3 cells, and it further declined after infection (Figure 8A). These findings suggest that VDAC3 is involved in redox metabolism. In addition, taurine and hypotaurine, which are well known for their antioxidant activity, changed in the infected cells. Levels of taurine, hypotaurine, and 3-sulfinoalanine (an intermediate involved in hypotaurine/taurine synthesis) decreased in Vero-NTC cells upon infection. However, there were significant increases in taurine, hypotaurine, and 3-sulfinoalanine in the infected Vero-shVDAC3 cells (Figure 8B–D). Moreover, the level of 6-phosphogluconate (6-PG), an intermediate of the pentose phosphate pathway, decreased in the infected Vero-NTC cells. The 6-PG level in Vero-shVDAC3 was elevated as compared with that of Vero-NTC cells. It remained elevated in these KD cells after infection. These findings are consistent with the notion that oxidative stress is induced in the infected cells. More important, silencing of *VDAC3* gene expression increases cellular antioxidative capacity, especially in the infected cells. This implies the possible involvement of VDAC3 in negative regulation of antioxidant metabolism. 

Of the aforementioned antioxidant molecules, hypotaurine is particularly interesting. Hypotaurine is a precursor to taurine, and has significantly higher total oxy-radical scavenging capacity than the latter [41]. We examined whether a change in hypotaurine alters the outcome of viral replication. As shown in Figure S3, both co-treatment and post-treatment of EV71-infected cells with hypotaurine substantially inhibited viral replication. Hypotaurine treatment effectively abated the virus-induced mitochondrial ROS formation (Figure S4). Hypotaurine was not cytotoxic at the concentration used (Figure S1B). We further studied if the 2B-VDAC3 interaction negatively regulates the hypotaurine formation. As shown in Figure S5, exogenous GFP-2B expression caused a significant reduction in hypotaurine level in Vero-NTC cells. In contrast, knockdown of *VDAC3* gene expression enhanced hypotaurine formation, suggestive of a regulatory role of VDAC3 in control of hypotaurine formation. These findings imply that the virus-induced dysregulation of hypotaurine/taurine metabolism, which affects redox homeostasis and viral replication, may be functionally associated with the 2B-VDAC3 interaction. 

## 4. Discussion

Our present study demonstrates that 2B specifically bound mitochondrial protein VDAC3, and such interaction was found at specific locations on the periphery of mitochondria in cells expressing these proteins. Silencing *VDAC3* gene or Mito-TEMPO treatment diminished the mitochondrial ROS generation induced by EV71 infection and 2B expression, and was inhibitory to viral replication. Moreover, *VDAC3* KD was associated with enhancement of the antioxidative pathway, such as hypotaurine/taurine synthesis, in the infected cells. Taken together, these findings advocate the notion that VDAC3 interacts with 2B to elicit mitochondrial ROS generation and modulates the antioxidant metabolism, which is conducive to viral replication. 

Infection with a number of viruses causes mitochondrial anomalies. Human herpesviruses [42], parvovirus [43], and Mink enteritis virus [44] cause mitochondrial depolarization and increase mitochondrial ROS production. Likewise, hepatitis B and C virus [45,46,47], rabies virus [48], respiratory syncytial virus [49], and rubella virus [50,51] disrupt the electron transport chain, leading to enhanced superoxide production. Other viruses, including dengue virus [52], rhinovirus [53] and human papillomavirus [54], elicit mitochondrial dysfunction and ROS release. Our findings suggest that EV71 falls into the class of viruses that cause mitochondrial dysfunction and ROS generation. 

The roles of ROS are either suppressive on, or promotional to, viral infection and pathogenesis. Mitochondrial ROS are implicated in mitochondrial antiviral signaling (MAVS). ROS are essential to RIG-1-mediated antiviral immunity for their involvement in modulation of *MAVS* expression [55] and MAVS oligomerization [56]. On the contrary, respiratory syncytial virus infection is associated with increased mitochondrial ROS production, which promotes viral replication [57]. Intracellular ROS enhances influenza A virus replication and inhibits antiviral immune responses [58,59]. Virus-induced ROS promote EV71 replication in a positive feedback manner [10]. This raises the possibility that antioxidants may have therapeutic potential. We have shown that treatment with mitochondrion-specific antioxidant Mito-TEMPO substantially inhibited EV71 replication. Mito-TEMPO and another antioxidant mitoQ provide effective protection against viruses such as dengue virus [52] and influenza virus [60]. 

Our co-immunoprecipitation-proteomic study revealed that 2B interacts with a number of host cell proteins. These include proteins of various respiratory complexes and VDAC3. The specific interaction between VDAC3 and 2B was validated by the reciprocal immunoprecipitation approach and use of proper negative controls (Figure 3). Furthermore, we employed a confocal microscopic approach to demonstrate that 2B and VDAC3 co-localizes at discrete locations on the periphery of mitochondria (Figure 4). VDAC3 is located in mitochondrial outer membrane [24], while 2B is embedded in the membranes of ER and Golgi body [28,61]. The discrete locations where VDAC3 and 2B co-localizes may represent the ER-mitochondrion or Golgi-mitochondrion contact points. It is known that VDAC1 interacts with glucose-regulated protein 75 and IP3R at mitochondria-associated membranes (MAMs) [62]. It is possible that VDAC3 binds to 2B in an analogous way. MAMs represent the contact points between ER and mitochondria. They are dynamic structures that facilitate the interorganellar exchange of lipids, calcium ions, and ROS, and may be implicated in signaling, autophagy, and regulation of ER stress and immunity [63]. The Golgi-mitochondrion contact point may have similar roles, such as lipid transport [64]. It is plausible that 2B may bind to VDAC3 at the MAMs or Golgi-mitochondrion contact points and regulate their functions in the infected cells.

Interaction between 2B and VDAC3 may have functional implications. As VDAC is involved in calcium ion transport between ER and mitochondria [65], it is possible that 2B might dysregulate the VDAC3-mediated calcium transport. However, there was no difference in the infection-induced changes in calcium ion levels between control and *VDAC3* knockdown cells (our unpublished data), suggesting that the calcium ion signaling is not involved in ROS generation. VDAC acts as transporter of metabolites such as nucleotides, dinucleotides, pyruvate, malate, succinate, etc. [66,67,68]. It has also been shown that VDAC controls production and release of superoxide anion (O_2_^·−^). Treatment with 4-diisothiocyano-2,2-disulfonic acid stilbene, a VDAC inhibitor, substantially inhibited superoxide anion formation [69]. It is thought that VDAC regulates the mitochondrial import of respiratory substrates which are further metabolized via tricarboxylic acid cycle. Electron pairs from nicotinamide adenine dinucleotide (NADH) flow down the electron transport chain, which is at least partially disrupted in the infected cells [20], to enhance superoxide anion production. The superoxide anion can be released to cytosol through VDAC, or metabolized to hydrogen peroxide by matrix-localized manganese superoxide dismutase [70]. Hydrogen peroxide can pass through membranes. Booth et al. proposed the existence of hydrogen peroxide nanodomains close to the ER-mitochondrion contact points [71]. It is likely that EV71 2B interacts with VDAC3 to alter its channel activity and metabolism, and to promote ROS formation in this manner. It is not unprecedented. Hepatitis B virus X protein binds to VDAC3 to dysregulate mitochondrial metabolism [72]. The ability of protein–protein interaction to modulate the opening and closure of VDAC is exemplified by the interaction between free tubulin and VDAC1 [73]. 

The increase in oxidative stress in EV71-infected cells is accompanied by decreases in abundance of antioxidants. More specifically, taurine and hypotaurine diminished (Figure 8). Silencing of *VDAC3* expression robustly increased their levels, especially after infection. This correlates with the reduction in virus-induced ROS generation. Viral infection may suppress hypotaurine/taurine synthesis. Consistent with this, exogenous 2B expression has been found to reduce the hypotaurine abundance (Figure S5). However, VDAC3 deficiency increased the hypotaurine levels in the control vector- and 2B expression vector-transfected cells. It is probable that the 2B-VDAC3 interaction might inhibit expression of the enzymes involved in hypotaurine metabolism, while reduction in VDAC3 expression led to a relief from such inhibitory effect. Reina et al. reported that VDAC3 deficiency is associated with expression of antioxidative proteins, including superoxide dismutase and thioredoxin [74]. Previous study has shown that VDAC3 interacts with a number of proteins, including peroxiredoxin 2 and 6, translationally controlled tumor protein (TCTP), oxygen-regulated protein, etc. [75]. Under the condition of VDAC3 deficiency, the VDAC3-interacting proteins may be present in the free form. Peroxiredoxin can bind to FOXO and regulate its further phosphorylation and nuclear translocation via disulfide exchange [76,77]. FOXO is known to control the expression of antioxidative enzymes or proteins. Expression of cysteine dioxygenase, an enzyme involved in oxidation of cysteine to 3′-sulfinoalanine, is upregulated in the *FOXO1*-trangenic mice [78]. Moreover, TCTP enhances the activity of peroxiredoxin 1 through inhibition of proteolysis and inactivating Mst1-mediated phosphorylation [79]. There exists a possibility that the 2B-VDAC3 interaction may stabilize the binding of peroxiredoxin and modulate the redox signaling. This may suppress the expression of antioxidative enzymes, such as those involved in hypotaurine synthesis. 

Hypotaurine and taurine are chemically 2-amino derivatives of ethanesulfinic acid and ethanesulfonic acid. A major pathway for synthesis of taurine involves the cysteine dioxygenase-catalyzed formation of 3-sulfino-L-alanine from cysteine, and its subsequent conversion to hypotaurine by cysteine sulfinic acid decarboxylase. Enzymatic or non-enzymatic oxidation of hypotaurine yields taurine [80]. Both hypotaurine and taurine are known to act as antioxidants. Previous studies showed that taurine offers a protective effect against oxidative stress in mitochondria [81]. Though not a typical radical scavenger, taurine can capture hypochlorous acid [82]. Taurine has been reported to increase the protein level and activity of copper zinc superoxide dismutase [83], and to stabilize the GSH pool in response to toxic insult [84]. Hypotaurine is superior to taurine in terms of its scavenging capacity [41]. It reacts efficiently with superoxide anion, hydroxyl radical, and hydrogen peroxide [82]. We previously showed that the antiviral activities of natural compounds correlate with their antioxidant capacities [85]. Exogenous hypotaurine treatment effectively inhibited viral replication (Figure S3). Our findings imply that enterovirus-induced repression of antioxidative metabolic pathway, such as hypotaurine/taurine synthesis, may promote viral infection. 

It is intriguing that ROS favors viral replication. Redox modification of viral proteins may alter their activities. The guanylyltransferase activity of flaviviral NS5 capping enzyme is enhanced by oxidants, which increases its capping activity and facilitates the genome replication [86]. Protein SOMOylation, which is inhibited under oxidizing condition [87], can be a regulatory factor. The SOMOylation of EV71 3C protein may promote its proteolysis [88]. It is possible that the degradation of 3C protein is blocked in the midst of an increased mitochondrial ROS generation. Besides, protein glutathionylation that is enhanced by ROS may modulate the activities of host cell or viral proteins [89]. 

In conclusion, the interaction between 2B and VDAC3 plays essential roles in ROS generation and modulation of antioxidative pathway(s). The resultant oxidative stress exerts a promotional effect on viral replication. 

## Data Availability

The data presented in this study are available upon request from the corresponding author. The data are not publicly available due to patent application request.

## Supplementary Materials

The following supporting information can be downloaded at: https://www.mdpi.com/article/10.3390/v14081717/s1, Figure S1: Mito-TEMPO and hypotaurine have no cytotoxicity at the concentrations used, Figure S2: Specific inhibitory effect of the shRNA against VDAC3 on viral replication, Figure S3: Hypotaurine treatment inhibits viral replication, Figure S4: Hypotaurine suppresses virus-induced mitochondrial ROS generation, Figure S5: 2B expression inhibits hypotaurine formation through its interaction with VDAC3, Table S1: Mitochondrial proteins differentially co-immunoprecipitated with 2B.
