# Supplementary material for: Enteroviral 2B Interacts with VDAC3 to Regulate Reactive Oxygen Species Generation That Is Essential to Viral Replication
Source: Viruses. 2022 Aug 4;14(8):1717. doi: 10.3390/v14081717 (PMC9416218; doi:10.3390/v14081717)
Supplement: Supplementary file 1 [file viruses-14-01717-s001.zip › Supplemental_Methods.pdf]

Supplemental Methods

Generation of the VDAC1 and TOMM70A knockdown cells

The lentiviral vectors encoding the shRNAs against *VDAC1* gene (TRCN0000278564) and *TOMM70A* gene (TRCN0000257097), and that encoding non-target control (NTC) shRNAs were acquired from RNA Technology Platform and Gene Manipulation Core Laboratory (Academia Sinica, Taipei). The lentiviral particles were packaged and Vero cells were transduced as described in the *Materials and Methods* section.

The reverse-transcription quantitative polymerase chain reaction (RT-qPCR) was performed as described in the *Materials and Methods* section. The forward primer for quantification of *VDAC1* expression was 5′-GGGTGCTCTGGTGCTAGGT-3′; the reverse primer used was 5′-GACAGCGGTCTCCAACTTCT-3′. The forward primer for quantification of *TOMM70A* expression was 5′-CTCTGGCACAAGCACAGAAA-3′; the reverse primer used was 5′-CTGTTAATGCCTGGGCGTAT-3′.

Fluorometric determination using CLARIOstar Plus microplate reader

Vero cells were seeded at a density of 2×10^5 cells per well in the microplate, and were mock- or infected with EV71 at an MOI of 1.25 for 1 h. They were un-, co-, or post-treated with hypotaurine. After 24 h, cells were stained with MitoSOX Red at 37 °C for 30 min in a humidified atmosphere of 5% carbon dioxide. The fluorescence was measured using CLARIOstar Plus microplate reader (BMG LABTECH GmbH; Ortenberg, Germany). The microplate was scanned with excitation wavelength set at 510 nm and emission wavelength set at 580 nm.
