# Supplementary material for: Enteroviral 2B Interacts with VDAC3 to Regulate Reactive Oxygen Species Generation That Is Essential to Viral Replication
Source: Viruses. 2022 Aug 4;14(8):1717. doi: 10.3390/v14081717 (PMC9416218; doi:10.3390/v14081717)
Supplement: Supplementary file 1 [file viruses-14-01717-s001.zip › Supplementary_Figures.pdf]

Supplementary Figures

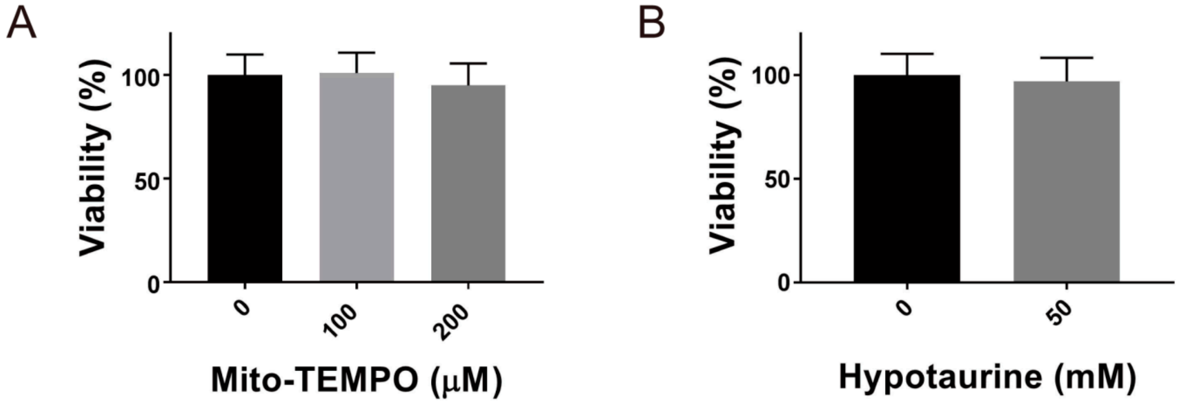

Figure S1. Mito-TEMPO and hypotaurine have no cytotoxicity at the concentrations used. Vero cells were treated with the indicated concentrations of Mito-TEMPO (A) and hypotaurine (B) for 24 h. Cell viability (*Viability*) was determined by cell counting. The results are mean ± SD, n = 3.

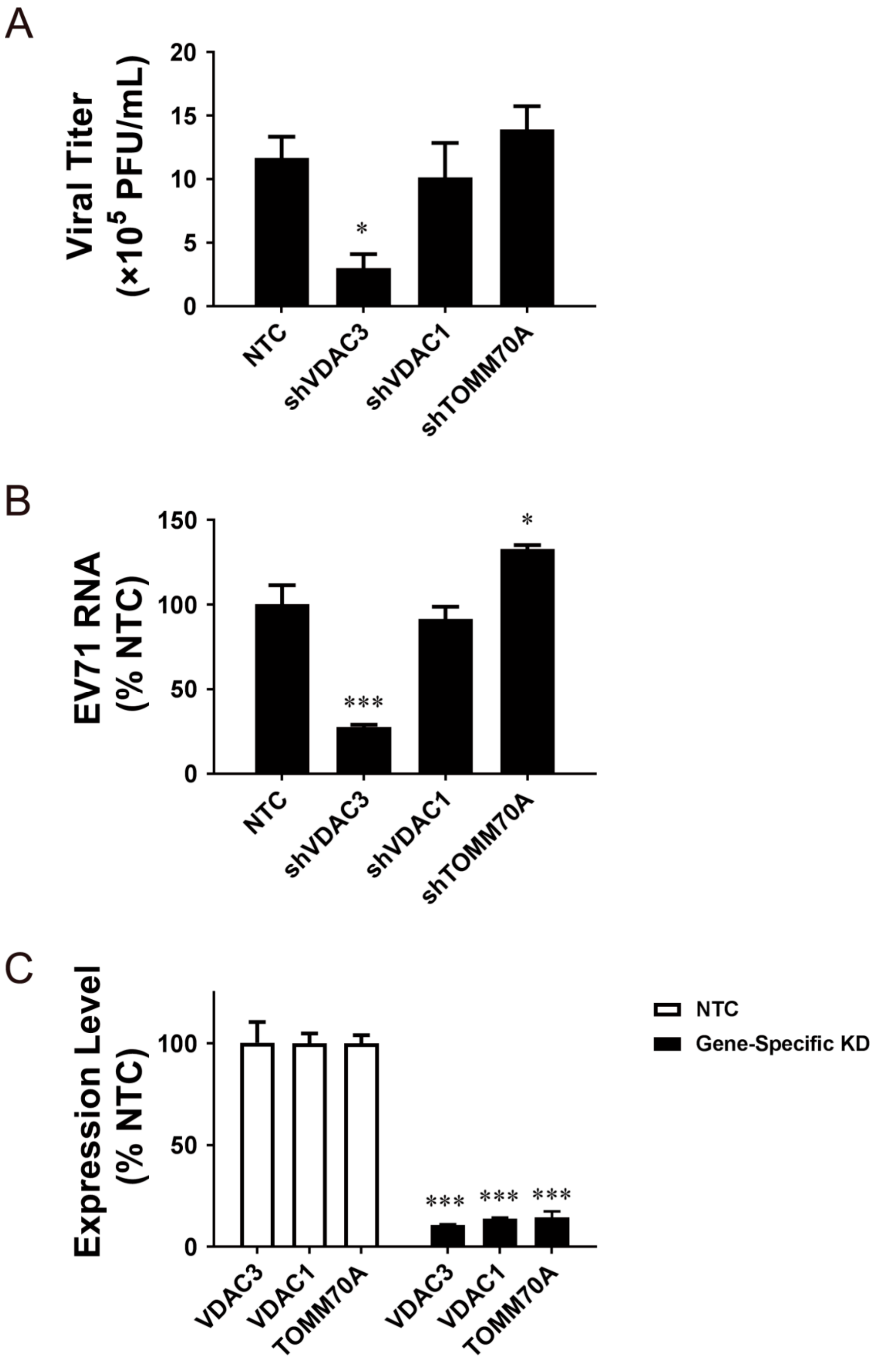

Figure S2. Specific inhibitory effect of the shRNA against VDAC3 on viral replication. Vero cells were transduced with lentiviral vectors encoding control shRNA (*NTC*) or the shRNAs against VDAC3 (*shVDAC3*), VDAC1 (*shVDAC1*) and TOMM70A (*shTOMM70A*). The transduced cells were infected with EV71 at an MOI of 10, and the titer (A) and genomic RNA level (B) of progeny virus were determined at 6 h post-infection. The titer is expressed in plaque-forming units (PFU) per mL. The level of EV71 genomic RNA is expressed as the percentage of that of infected-NTC cells. (C) The levels of *VDAC3*, *VDAC1* and *TOMM70A* transcripts in NTC and knockdown (*KD*) cells were quantified by qRT-PCR. The level of each transcript is expressed as the percentage of that of NTC cells. The results are mean ± SD, n = 6. $^{*}p < 0.05$, $^{***}p < 0.005$ vs. NTC cells.

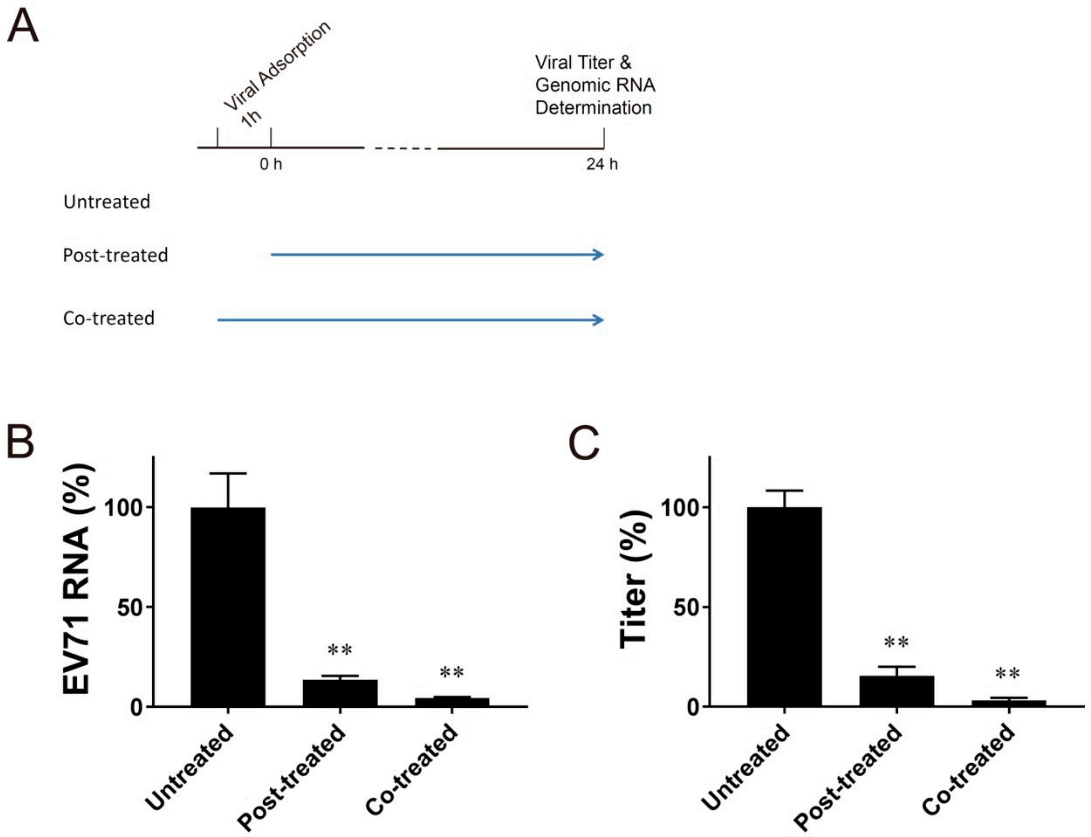

Figure S3. Hypotaurine treatment inhibits viral replication. Vero cells were mock or infected with EV71 at an MOI of 1.25 for 1 h, and were co- or post-treated with 50 mM hypotaurine (as indicated by blue line) (A). Twenty-four hours later, the level of viral genomic RNA (B) and the titer of progeny virus (C) were determined. The results are expressed as the percentages of those of untreated cells. The results are mean ± SD, n = 6. **p < 0.01 vs. the untreated EV71-infected cells.

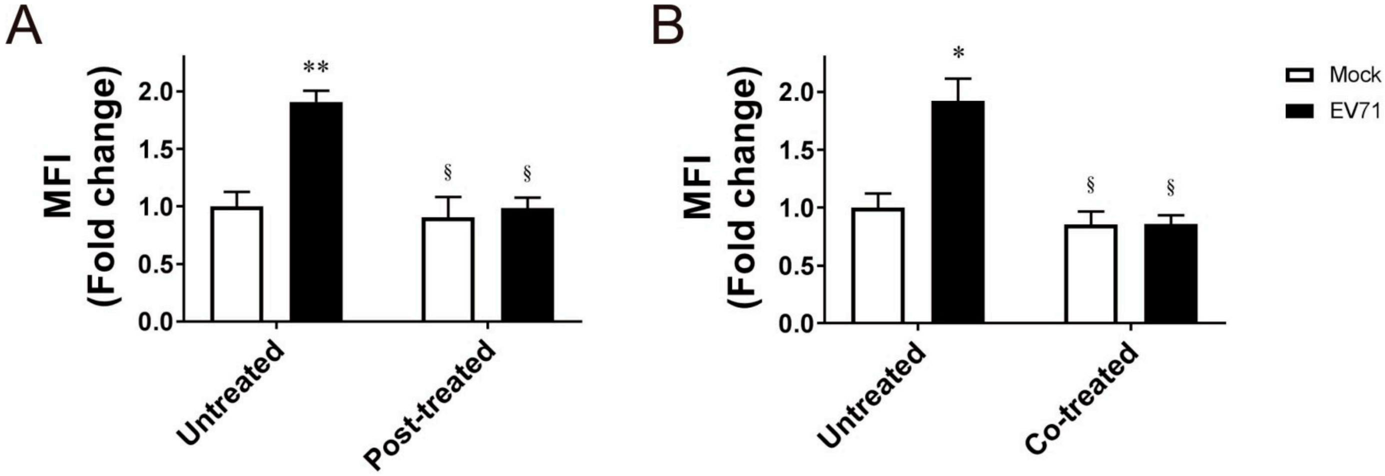

Figure S4. Hypotaurine suppresses virus-induced mitochondrial ROS generation. Vero cells were mock or infected with EV71 at an MOI of 1.25, and post- (A) or co-treated (B) with 50 mM hypotaurine. Twenty-four hours later, the cells were stained with MitoSOX red and mitochondrial ROS level was determined using CLARIOstar Plus multi-mode microplate reader as described in *Supplemental Methods*. The MFI is expressed as fold change relative to that of untreated mock-infected cells. The results are mean ± SD, n = 6. *p < 0.05, **p < 0.01 vs. untreated mock-infected cells; §p <0.05 vs. untreated EV71-infected cells.

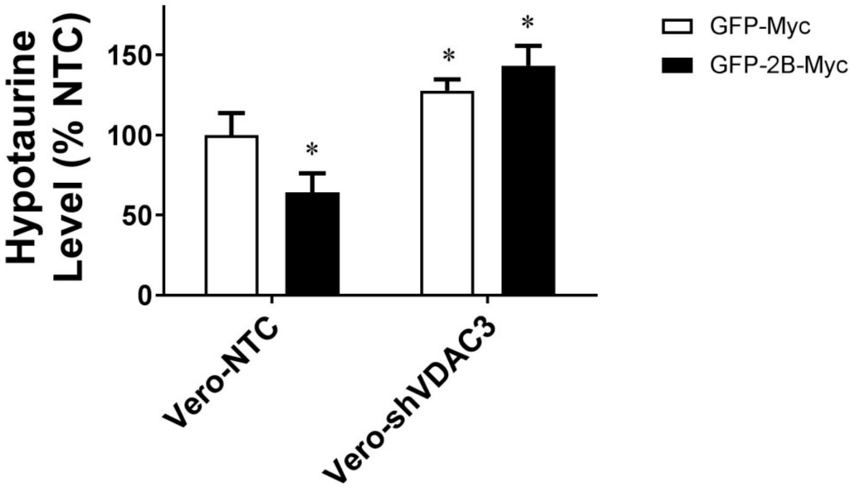

Figure S5. 2B expression inhibits hypotaurine formation through its interaction with VDAC3. Vero-NTC and Vero-shVDAC3 cells were transfected with the GFP-2B-Myc or GFP-Myc expression vector. Forty-eight hours later, the cells were harvested for analysis of hypotaurine. Its level is expressed as percentage of that of the GFP-Myc-expressing Vero-NTC cells. Data are mean ± SD (n = 9). *$p < 0.05$, vs. the GFP-Myc-expressing Vero-NTC cells.
