# Supplementary material for: Enteroviral 2B Interacts with VDAC3 to Regulate Reactive Oxygen Species Generation That Is Essential to Viral Replication
Source: Viruses. 2022 Aug 4;14(8):1717. doi: 10.3390/v14081717 (PMC9416218; doi:10.3390/v14081717)
Supplement: Supplementary file 1 [file viruses-14-01717-s001.zip › Table_S1.pdf]

Table S1. Mitochondrial proteins differentially co-immunoprecipitated with 2B

Symbol	Entrez Gene Name	Protein Accession Number	Heavy/Light Ratio	Heavy/Light Count
SDHB	succinate dehydrogenase complex iron sulfur subunit B	P21912	**1183.487**	6
UQCRB	ubiquinol-cytochrome c reductase binding protein	P14927	**966.47**	3
ATP5MG	ATP Synthase Membrane Subunit G	O75964	**848.136**	2
UQCRC1	ubiquinol-cytochrome c reductase core protein 1	P31930	**597.584**	9
VDAC3	voltage-dependent anion channel 3	Q9Y277	**548.569**	2
NDUFS1	NADH:ubiquinone oxidoreductase core subunit S1	P28331	**478.214**	4
ATP5PF	ATP synthase peripheral stalk subunit F6	P18859	**331.494**	4
NDUFV2	NADH:ubiquinone oxidoreductase core subunit V2	P19404	**320.749**	2
NDUFA7	NADH:ubiquinone oxidoreductase subunit A7	O95182	**220.892**	3
ATP5PD	ATP Synthase Peripheral Stalk Subunit D	O75947	**206.964**	6

Symbol refers to the official symbol of the protein-coding gene; Entrez gene name refers to the full gene name provided by Entrez database; protein accession number is an unique identifier assigned to the protein by the UniProtKB/Swiss-Prot database; the heavy/light ratio refers to the ratio of the quantification values of the heavy (i.e. deuterated) and light (i.e. non-deuterated) quantification channels; heavy/light count refers to the number of peptide ratios used for calculation of a particular protein ratio.
